# Glucose Intolerance and Colorectal Cancer Risk in a Nested Case-Control Study among Japanese People

**DOI:** 10.2188/jea.15.S180

**Published:** 2005-08-18

**Authors:** Kotaro Ozasa, Yoshinori Ito, Koji Suzuki, Yoshiyuki Watanabe, Masayo Kojima, Sadao Suzuki, Shinkan Tokudome, Koji Tamakoshi, Hideaki Toyoshima, Miyuki Kawado, Shuji Hashimoto, Norihiko Hayakawa, Kenji Wakai, Akiko Tamakoshi

**Affiliations:** 1 Department of Epidemiology for Community Health and Medicine, Kyoto Prefectural University of Medicine Graduate School of Medical Science.; 2 Department of Public Health, Fujita Health University School of Health Sciences.; 3 Department of Health Promotion and Preventive Medicine, Nagoya City University Graduate School of Medical Sciences.; 4 Department of Public Health/Health Information Dynamics, Nagoya University Graduate School of Medicine.; 5 Department of Hygiene, Fujita Health University School of Medicine.; 6 Department of Epidemiology, Research Institute for Radiation Biology and Medicine, Hiroshima University.; 7 Division of Epidemiology and Prevention, Aichi Cancer Center Research Institute.; 8 Department of Preventive Medicine/Biostatistics and Medical Decision Making, Nagoya University Graduate School of Medicine.

**Keywords:** Glucose Intolerance, glycoalbumin, Colorectal Neoplasms, Cohort Studies, Epidemiology

## Abstract

BACKGROUND: Glucose intolerance may increase the risk of developing colorectal cancer.

METHODS: In a sero-epidemiological nested case-control study, conducted as part of the Japan Collaborative Cohort Study (JACC Study) for Evaluation of Cancer Risk, we measured serum glycoalbumin in 123 patients with colorectal cancer and 279 controls. Conditional logistic regression was used to evaluate the risk of colorectal cancer.

RESULTS: There were trends towards an association between high levels of glycoalbumin and an increased risk of colorectal cancer in men (odds ratio [OR] = 2.39; 95% confidence interval [CI]; 0.89-6.36) and between high levels of glycoalbumin and a decreased risk of colorectal cancer in women (OR = 0.41; 95% CI, 0.14-1.04).

CONCLUSIONS: A high level of glycoalbumin may increase the risk of colorectal cancer in men. The finding that high levels of glycoalbumin in women decreased their risk of colorectal cancer was inconsistent with previous reports, and may have been the result of limitations in the procedure in selecting samples and statistical power.

Glucose intolerance or an elevated serum concentration of insulin is thought to be a risk factor for the development of colorectal cancer.^1^^-^^7^ This association is based on the hypothesis that hyperinsulinemia promotes colon carcinogenesis.^1^ In vitro, insulin acts as a growth factor for colonic epithelial cells and a mitogen for tumor cell growth.^1^ The incidence rates of glucose intolerance and diabetes mellitus are increasing in Japan, and this, together with increased fat intake and sedentary working conditions, may be associated with the recently observed increase in the incidence of colorectal cancer and death from this disease in Japan. However, no prospective epidemiological study of the association between these biomarkers and colon cancer risk has been performed in the general population in Japan.

To determine the association between glucose intolerance and colorectal cancer in Japan, we carried out a nested case-control study using stored sera from the Japan Collaborative Cohort Study (JACC Study) for Evaluation of Cancer Risk sponsored by the Ministry of Education, Science, Sports and Culture of Japan (Monbusho).

## METHODS

The general aspects of the study population, procedures for conducting the baseline survey using a self-administered questionnaire, collecting serum samples, and following-up in the JACC study have been described previously.^8^ Diagnosis of colorectal cancer was defined by codes C18, C19, and C20, in the International Statistical Classification of Diseases and Related Health Problems, 10th Revision.^9^ Subjects were followed until the end of 1999 for death in all 45 study areas and until the end of 1997 for incidence in the 24 study areas in which incident cases were surveyed using a cancer registry, but follow-up was censored earlier in some areas.

We identified 220 incidents or deaths from colorectal cancer in the study subjects who donated serum samples, who did not have colorectal cancer and who were not being treated for diabetes mellitus at the time of the baseline survey. For each of these 220 patients, we randomly selected two or three control individuals, matched for study area, sex and age, from the study subjects who remained alive and cancer free (602 controls). We could not measure serum glycoalbumin in all eligible cases and controls due to a lack of sufficient volume of serum from some of the subjects. Consequently, glycoalbumin was measured in 117 cases and 263 controls (samples from 51 subjects who died from colon cancer originated from all study areas and samples from 91 who died from or were diagnosed with colorectal cancer were from the 24 above-described study areas). The procedure used in this study to select the measured samples for glycoalbumin among primarily detected cases of colorectal cancer was not as stringent as that used in regular epidemiological studies, and the statistical power was limited by an insufficient number of samples, limiting the interpretation of the results of this analysis.

Serum concentrations of glycoalbumin were determined in 2003 using autoanalyzers (JCA-BM12, JEOL Ltd., Akishima, Japan) at SRL laboratory (Hachioji, Japan), with those performing the measurement blinded to the case-control status of samples. The coefficient of variance was 0.5-0.8% for glycoalbumin. Fasting status at the time of blood donation was not recorded.

The difference in means between cases and controls was examined by t-test, and that in proportion was examined by chi-square test. The risk of colorectal cancer was evaluated by odds ratios (ORs) and 95% confidence intervals (CIs) estimated in the conditional logistic model. The ORs were computed according to tertiles of assayed substances among controls by sex. ORs were further adjusted for potential confounders such as serum total cholesterol, body mass index (BMI), walking habits in daily life, and history of diabetes mellitus. Linear trends of ORs were examined using the medians of tertiles in the conditional logistic model. Calculations were conducted using SAS^®^ software (SAS Institute, Cary, NC) in the Academic Center for Computing and Media Studies, Kyoto University.

Individual written or oral consent, or consent from community representatives, was obtained, or a poster notification/opting-out system was applied.^8^ The Ethical Boards of Nagoya University School of Medicine and Fujita Health University approved this study.

## RESULTS

The mean ± standard deviation ages at baseline were 60.8 ± 8.4 years of age in male cancer cases, 60.0 ± 8.4 years in male controls, 61.1 ± 7.4 years in female cancer cases, and 60.7 ± 7.1 years in female controls. The distributions of serum glycoalbumin and total cholesterol concentrations, as well as BMI, are shown in Table 1. The mean concentrations of those substances did not differ between cases and controls. The distributions of walking habits in daily life and history of diabetes mellitus are shown in Table 2.

**Table 1.** Distribution of measured variables.

		Cases				Controls			
	
		No.	Median		(25, 75 percentile)	No.	Median	(25, 75 percentile)	p
Males									
Glycoalbumin	%	58	15.9	( 14.5, 17.8)		133	15.5	( 14.2, 17.4)	0.58
Total cholesterol	mg/dL	58	193	( 170, 215)		133	189	( 163, 206)	0.29
Body mass index		58	22.6	( 20.9, 24.6)		133	22.7	( 21.2, 24.8)	0.75
Females									
Glycoalbumin	%	59	15.4	( 14.3, 16.8)		130	15.7	( 15.0, 16.9)	0.33
Total cholesterol	mg/dL	59	201	( 181, 240)		130	209	( 186, 227)	0.77
Body mass index		59	23.7	( 21.6, 25.7)		130	23.0	( 21.4, 25.5)	0.42

P values were examined by t-test.

**Table 2.** Distribution of other potential confounding factors.

	Males			Females		
	
	Case	Control	p	Case	Control	p
Walking habit in daily life (/day)						
One hour or more	24	46		27	53	
30 minites to 1 hr	7	16		11	14	
Around 30 min	5	12		4	19	
Almost none	4	10	0.72	4	15	0.16

History of diabetes mellitus						
Yes	2	7		2	4	
No	54	122	0.59	48	122	0.78

P values were examined by chi-square test.

Data from subjects who did not respond or who were ineligible are not shown.

Table 3 shows the ORs and 95% CIs of colorectal cancer adjusted for matching variables - sex, age, and study area. High levels of glycoalbumin showed a trend towards association with an increased risk of colorectal cancer in men (OR = 2.39, p=0.081 for the highest tertile). The association seemed to be stronger for cancer of the colon than of the rectum, although both sites had a similar tendency. In contrast, high levels of glycoalbumin showed a trend towards association with a decreased risk of colorectal cancer in women (OR = 0.41, p=0.060 for the highest tertile). This tendency was similar for both colon and rectal cancer. Classified analyses by incidence of colon cancer and colon cancer deaths showed similar results (data not shown).

**Table 3.** Odds ratios (ORs) and 95% confidence intervals (CIs) of glycoalbumin.

	Males						Females					
	
	Category	Cases/ controls		OR	95% CI	p	Category	Cases/ controls		OR	95% CI	p
Colon and rectum (C18,C19,C20)	<14.6 (%)	15	47	1.00	0.66, 3.75	0.30	<15.2 (%)	27	47	1.00	0.40, 1.77	0.65
	14.6-16.2	18	46	1.57	0.89, 6.36	0.081	15.2-16.5	17	37	0.84	0.16, 1.04	0.060
	16.2+	25	25	2.39	Trend p=0.088		16.5+	15	46	0.41	Trend p=0.061	

Colon (C18)	<14.7 (%)	9	31	1.00	0.58, 5.36	0.31	<15.1 (%)	21	34	1.00	0.19, 1.28	0.15
	14.7-16.3	14	33	1.77	0.88, 10.3	0.076	15.1-16.3	10	33	0.50	0.26, 1.58	0.34
	16.3+	18	32	3.03	Trend p=0.082		16.3+	15	35	0.64	Trend p=0.28	

Rectum (C19,C20)	<14.5 (%)	6	13	1.00	0.20, 3.18	0.76	<15.6 (%)	7	11	1.00	0.14, 3.90	0.73
	14.5-16.2	5	15	0.81	0.19, 7.37	0.83	15.6-20.2	5	9	0.53	0.01, 1.64	0.11
	16.2+	7	13	1.21	Trend p=0.85		20.2+	1	10	0.28	Trend p=0.11	

Adjusting for total cholesterol, BMI, or walking habits in daily life did not substantially change the OR in either sex. Adjusting for history of diabetes mellitus slightly enhanced the association between glycoalbumin level and risk of colorectal cancer (OR = 0.39; 95% CI, 0.15-1.02) in females, but had little effect in males.

## DISCUSSION

Glycosylated protein levels reflect average glucose concentrations during a period that depends on the half-life of the protein. Glycoalbumin concentration is thought to reflect the average blood glucose concentration in the previous 1 to 2 weeks (half life=approximately 14 days), whereas glycohemoglobin A_1C_ (HbA_1C_) concentration is thought to reflect average blood glucose concentration during the previous 2 to 3 months (half life=120 days).^10^ Although an indicator that reflects long-term status is better for epidemiological studies, we assayed glycoalbumin because only serum samples were stored in the JACC Study.

Many epidemiologic studies have found an association between glucose intolerance and increased risk of colorectal cancer.^1^^-^^7^ Insulin is thought to influence colorectal carcinogenesis through its links with the insulin-like growth factors (IGFs) and IGF binding proteins (IGFBPs), which are overexpressed in many tumors.^6^ An insulin-associated decrease in IGFBP-1 and resultant increase in free IGF-1 may increase the risk of colorectal cancer.^1^^,^^6^ Thus, carcinogenesis is considered to be promoted by hyperinsulinemia. Glucose intolerance is not necessarily accompanied by hyperinsulinemia. However, it has been reported that elevated glucose or HbA1C levels were associated with colon cancer risk in Western countries.^2^^,^^3^^,^^6^^,^^7^ Hence we examined the association between glucose intolerance and colorectal cancer in Japanese people.

In this study, a high level of glycoalbumin was associated with an increased risk of colorectal cancer in men. The association with rectal cancer, however, was weak. These results appear to be in accord with the previous finding that glucose intolerance was more strongly associated with colon than with rectal cancer (the OR for the highest HbA_1C_ quartile relative to the lowest was 2.10 for proximal colon, 1.61 for distal colon, and 0.91 for rectum).^6^ Although a previous study has reported that a high blood glucose level increased the risk of colorectal cancer in women (RR = 1.98 for 8.0+ mM in non-fasting individuals) and an insignificant relative risk was shown in men (RR = 0.98),^3^ our study detected apparently diverse effects between sexes and a reduced risk for colon cancer in women with glucose intolerance.

The analysis of the whole cohort of the JACC Study indicated that a history of diabetes mellitus significantly increased the risk of colorectal cancer death in women (RR =1.70, 95% CI; 1.03-2.82), but did not increase the risk in men (RR = 0.85, 95% CI; 0.51-1.42) (unpublished data). As for the risk of colorectal cancer incidencce, RR = 1.19 (95% CI; 0.68-2.07) in men, and RR = 1.65 (95% CI; 0.88-3.10) in women. These findings are not consistent with the result of the present study; rather they are consistent with previous reports by other study groups.^2^^,^^3^^,^^6^^,^^7^ This inconsistency may be due to inadequate sample selection procedures and insufficient statistical power in the present work.

Adjustment for potential confounders, including serum total cholesterol, BMI, walking habits, and history of diabetes mellitus, had a minimal effect on our results.

In conclusion, a high level of glycoalbumin may increase the risk of colorectal cancer in men. The interpretation of the results was, however, limited by procedural inadequacies and insufficient statistical power.

## MEMBER LIST OF THE JACC STUDY GROUP

The present investigators involved, with the co-authorship of this paper, in the JACC Study and their affiliations are as follows: Dr. Akiko Tamakoshi (present chairman of the study group), Nagoya University Graduate School of Medicine; Dr. Mitsuru Mori, Sapporo Medical University School of Medicine; Dr. Yutaka Motohashi, Akita University School of Medicine; Dr. Ichiro Tsuji, Tohoku University Graduate School of Medicine; Dr. Yosikazu Nakamura, Jichi Medical School; Dr. Hiroyasu Iso, Institute of Community Medicine, University of Tsukuba; Dr. Haruo Mikami, Chiba Cancer Center; Dr. Yutaka Inaba, Juntendo University School of Medicine; Dr. Yoshiharu Hoshiyama, University of Human Arts and Sciences; Dr. Hiroshi Suzuki, Niigata University School of Medicine; Dr. Hiroyuki Shimizu, Gifu University School of Medicine; Dr. Hideaki Toyoshima, Nagoya University Graduate School of Medicine; Dr. Kenji Wakai, Aichi Cancer Center Research Institute; Dr. Shinkan Tokudome, Nagoya City University Graduate School of Medical Sciences; Dr. Yoshinori Ito, Fujita Health University School of Health Sciences; Dr. Shuji Hashimoto, Fujita Health University School of Medicine; Dr. Shogo Kikuchi, Aichi Medical University School of Medicine; Dr. Akio Koizumi, Graduate School of Medicine and Faculty of Medicine, Kyoto University; Dr. Takashi Kawamura, Kyoto University Center for Student Health; Dr. Yoshiyuki Watanabe, Kyoto Prefectural University of Medicine Graduate School of Medical Science; Dr. Tsuneharu Miki, Graduate School of Medical Science, Kyoto Prefectural University of Medicine; Dr. Chigusa Date, Faculty of Human Environmental Sciences, Mukogawa Women’s University ; Dr. Kiyomi Sakata, Wakayama Medical University; Dr. Takayuki Nose, Tottori University Faculty of Medicine; Dr. Norihiko Hayakawa, Research Institute for Radiation Biology and Medicine, Hiroshima University; Dr. Takesumi Yoshimura, Fukuoka Institute of Health and Environmental Sciences; Dr. Akira Shibata, Kurume University School of Medicine; Dr. Naoyuki Okamoto, Kanagawa Cancer Center; Dr. Hideo Shio, Moriyama Municipal Hospital; Dr. Yoshiyuki Ohno, Asahi Rosai Hospital; Dr. Tomoyuki Kitagawa, Cancer Institute of the Japanese Foundation for Cancer Research; Dr. Toshio Kuroki, Gifu University; and Dr. Kazuo Tajima, Aichi Cancer Center Research Institute.
